# Supplementary material for: Novel insights into the role of long non-coding RNA in the human malaria parasite, Plasmodium falciparum
Source: Nat Commun. 2023 Aug 22;14:5086. doi: 10.1038/s41467-023-40883-w (PMC10444892; doi:10.1038/s41467-023-40883-w)
Supplement: Supplementary file 1 — Supplementary Information [file 41467_2023_40883_MOESM1_ESM.pdf]

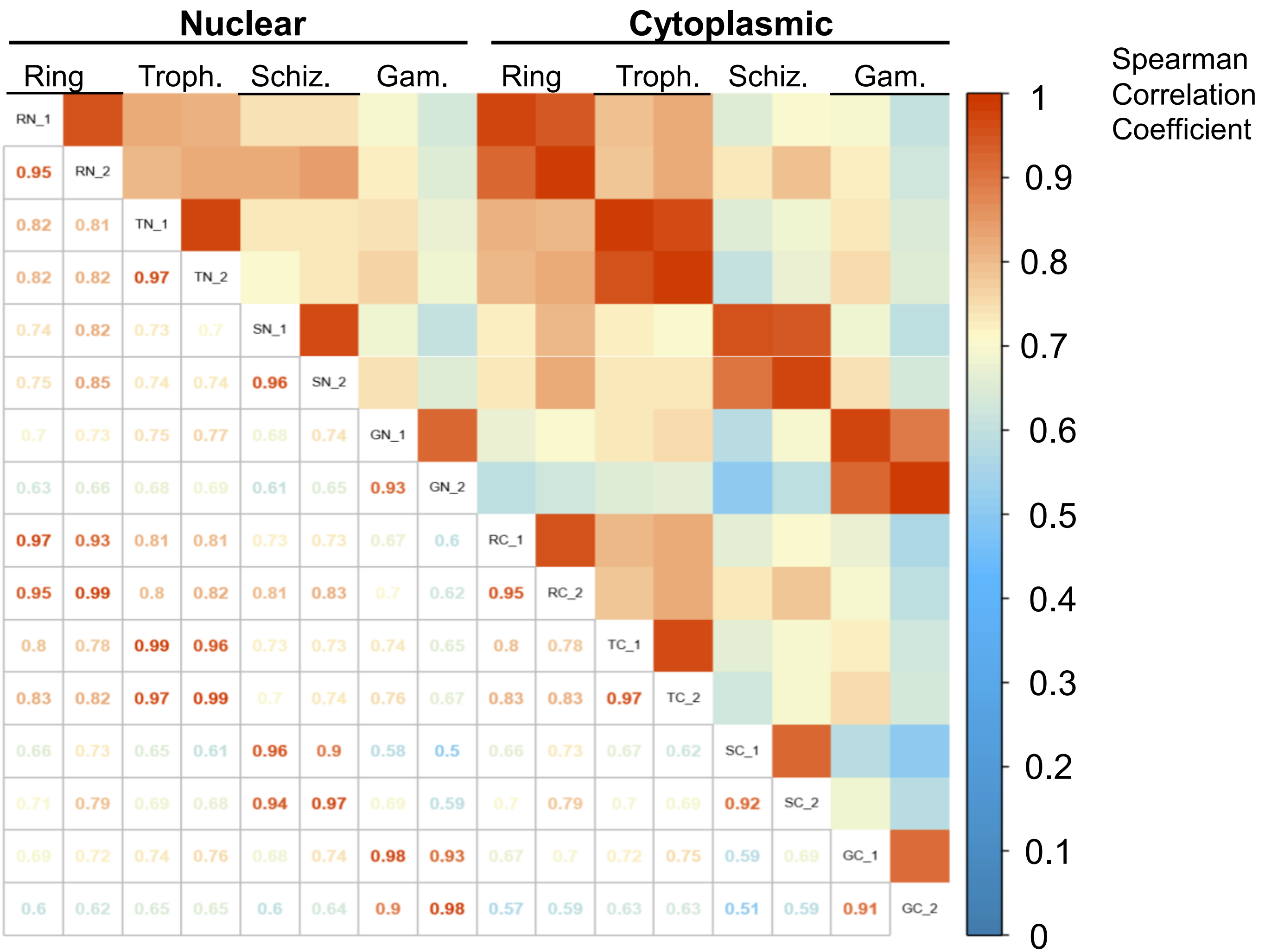

Fig. S1: Spearman correlation among gene expression levels of nuclear fraction and cytoplasmic fraction at Ring, Trophozoite (Troph.), Schizont (Schiz.) and Late Gametocyte (Gam) stages.

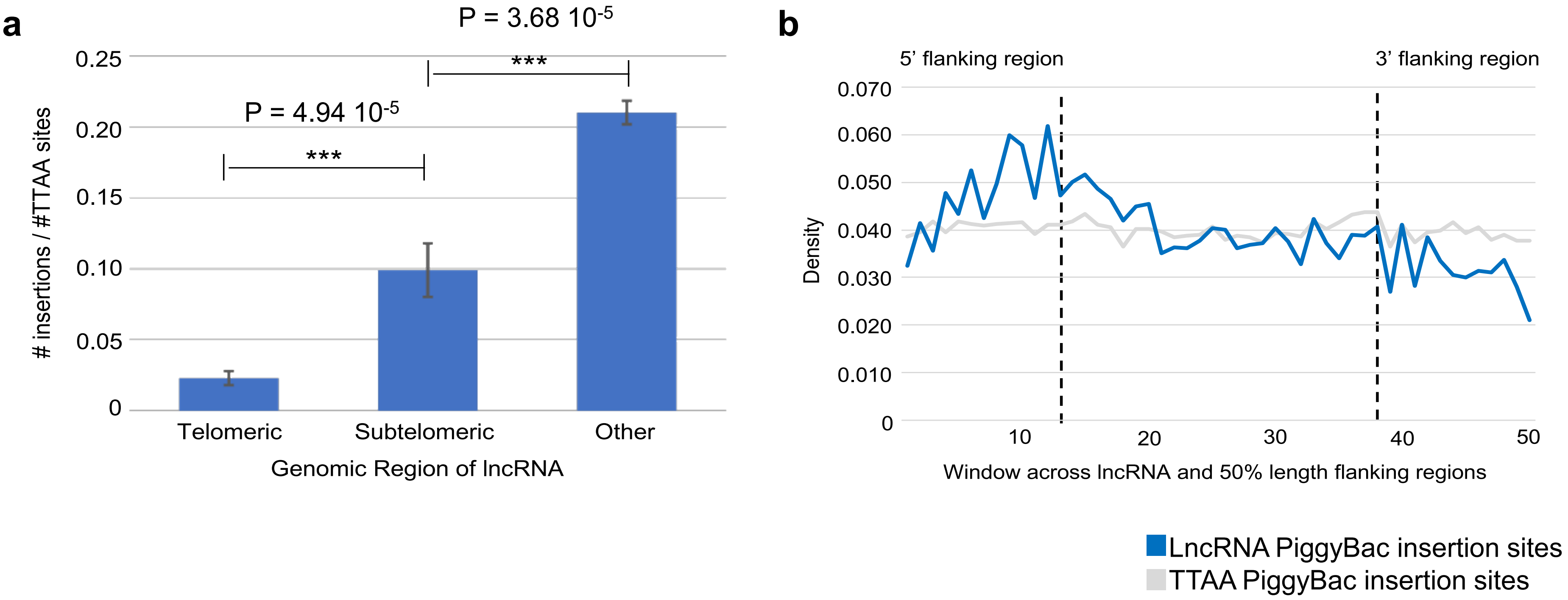

Fig. S2: Essentiality of lncRNAs in *P. falciparum*. (**a**) Number of piggyBac insertions per possible insertion site (TTAA site) for telomeric (n=51), subtelomeric (n=96), and other lncRNAs (n=1621). Telomeric lncRNAs have significantly fewest insertions/TTAA sites, followed by subtelomeric lncRNAs. Data are presented as mean values +/- SEM. P-values were calculated using two-tailed t-test. (**b**) Plot showing normalized density of piggyBac insertions and TTAA sites across all detected lncRNAs including flanking regions for each lncRNA, representing 50% of the lncRNA length on both the 5' and 3' sides.

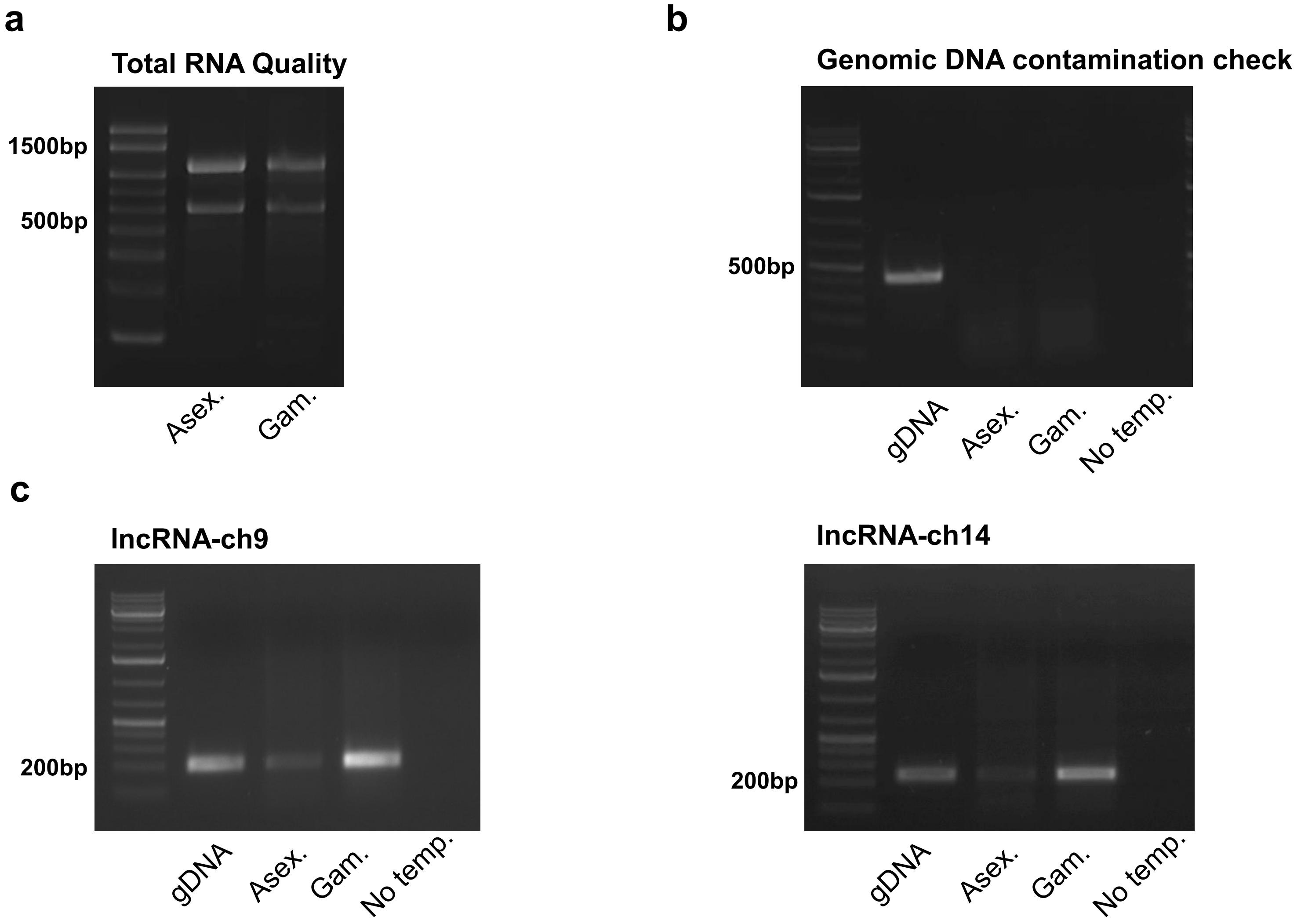

Fig. S3: Validation of gametocyte-specific lncRNAs expression (**a**) Total RNA was extracted from both asexual (Asex.) and gametocyte (Gam.) stage parasites. RNA quality was validated on agarose gel in two independent experiments. (**b**) Genomic DNA was removed and verified using reverse transcription polymerase chain reaction (RT-PCR) with primers designed to amplify a fragment of *pfAlba3* gene (PF3D7_1006200). Primers were designed on both sides of intron 1, yielding a 429 bp PCR product from genomic DNA and a 164 bp PCR product from cDNA. The absence of PCR product amplified from RNA confirms the absence of gDNA contamination. Two independent biological replicates were performed. (**c**) RT-PCR validation of two lncRNA-ch9 and lncRNA-ch14 that are most abundantly expressed at the gametocyte stage with high level of H3K9me3 mark. These gels are representative of two independent experiments. No temp.= No template.

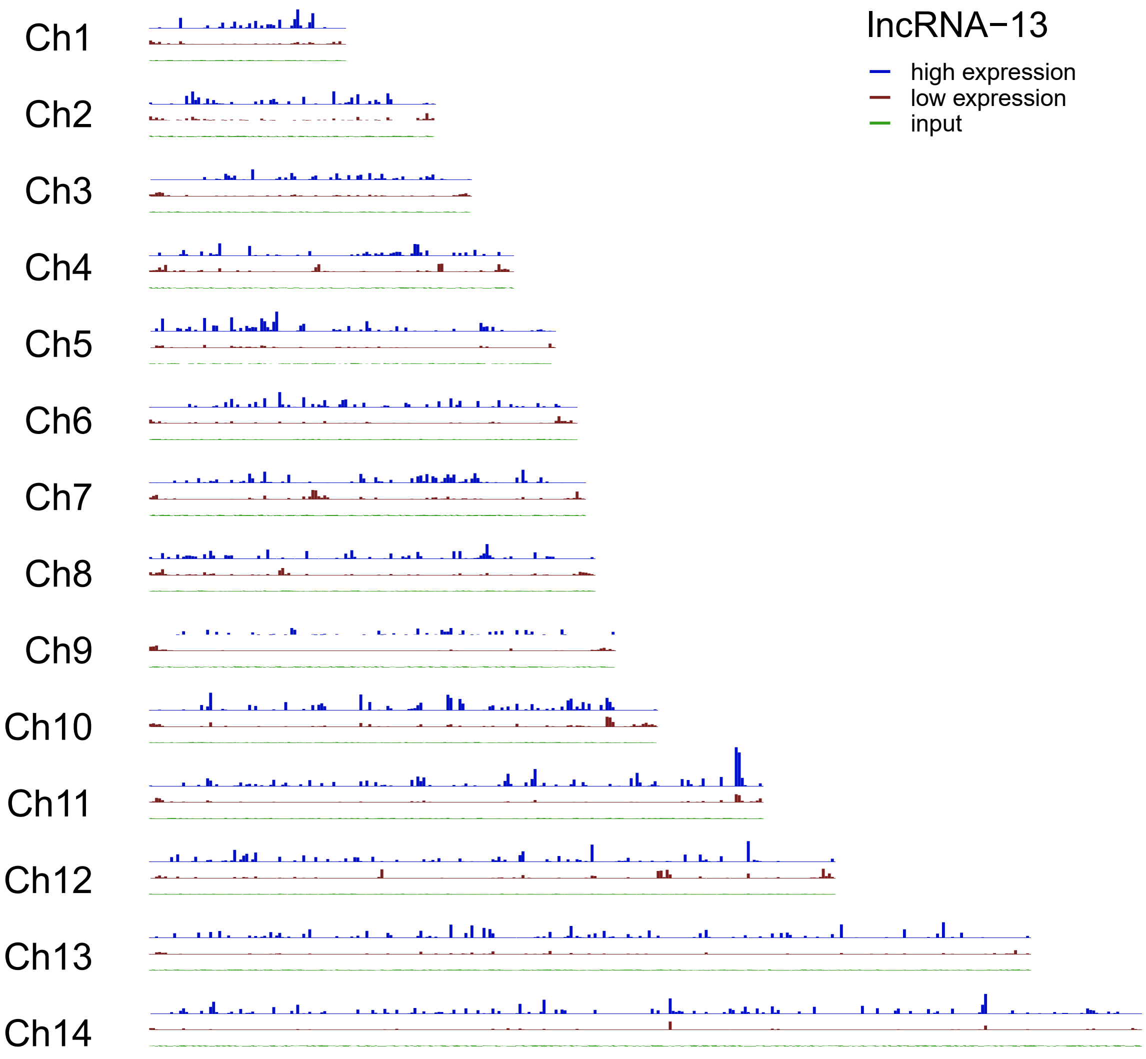

Fig. S4: (**a**) Genome wide ChIRP-seq signal for lncRNA-13. All lncRNA libraries, including the input, were normalized by dividing by the numbers of mapped reads in each of them. For each nucleotide, the signal from the input library was subtracted from each of the ChIRP-seq libraries, and any negative value was replaced with a zero. Genome tracks are displayed for each chromosome (Ch).

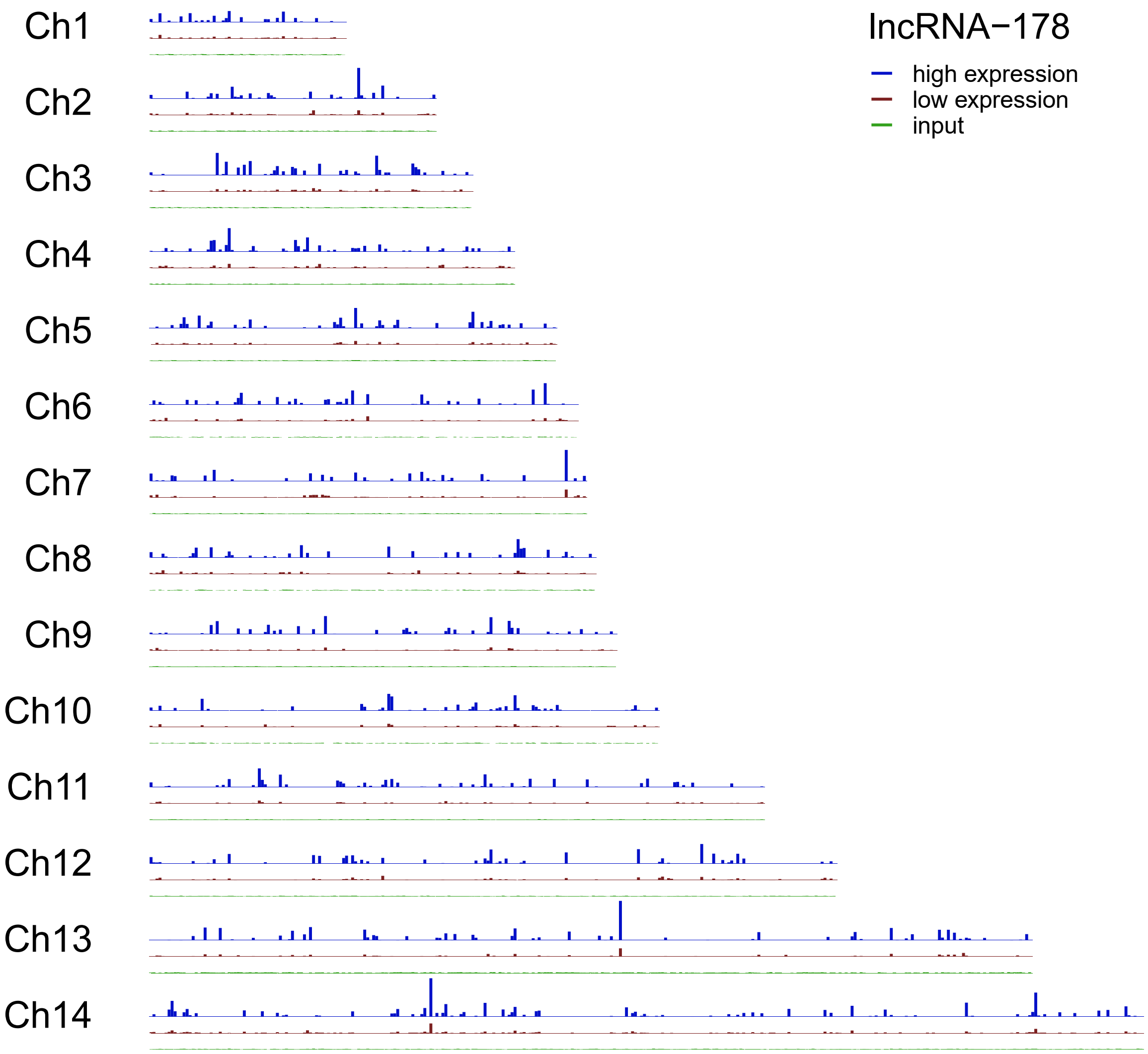

Fig. S4: (**a**) Genome wide ChIRP-seq signal for lncRNA-178. All lncRNA libraries, including the input, were normalized by dividing by the numbers of mapped reads in each of them. For each nucleotide, the signal from the input library was subtracted from each of the ChIRP-seq libraries, and any negative value was replaced with a zero. Genome tracks are displayed for each chromosome (Ch).

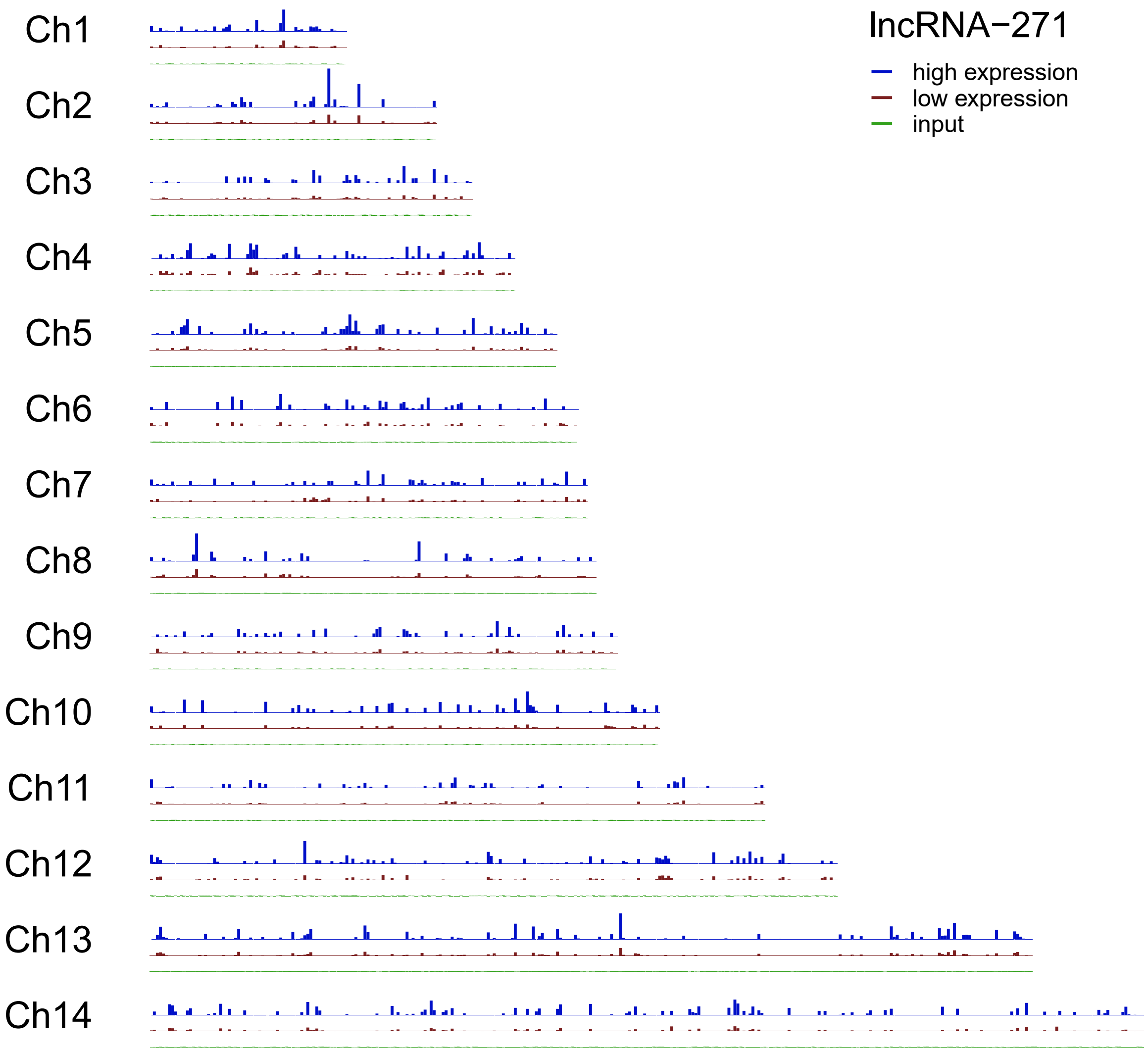

Fig. S4: (**a**) Genome wide ChIRP-seq signal for lncRNA-271. All lncRNA libraries, including the input, were normalized by dividing by the numbers of mapped reads in each of them. For each nucleotide, the signal from the input library was subtracted from each of the ChIRP-seq libraries, and any negative value was replaced with a zero. Genome tracks are displayed for each chromosome (Ch).

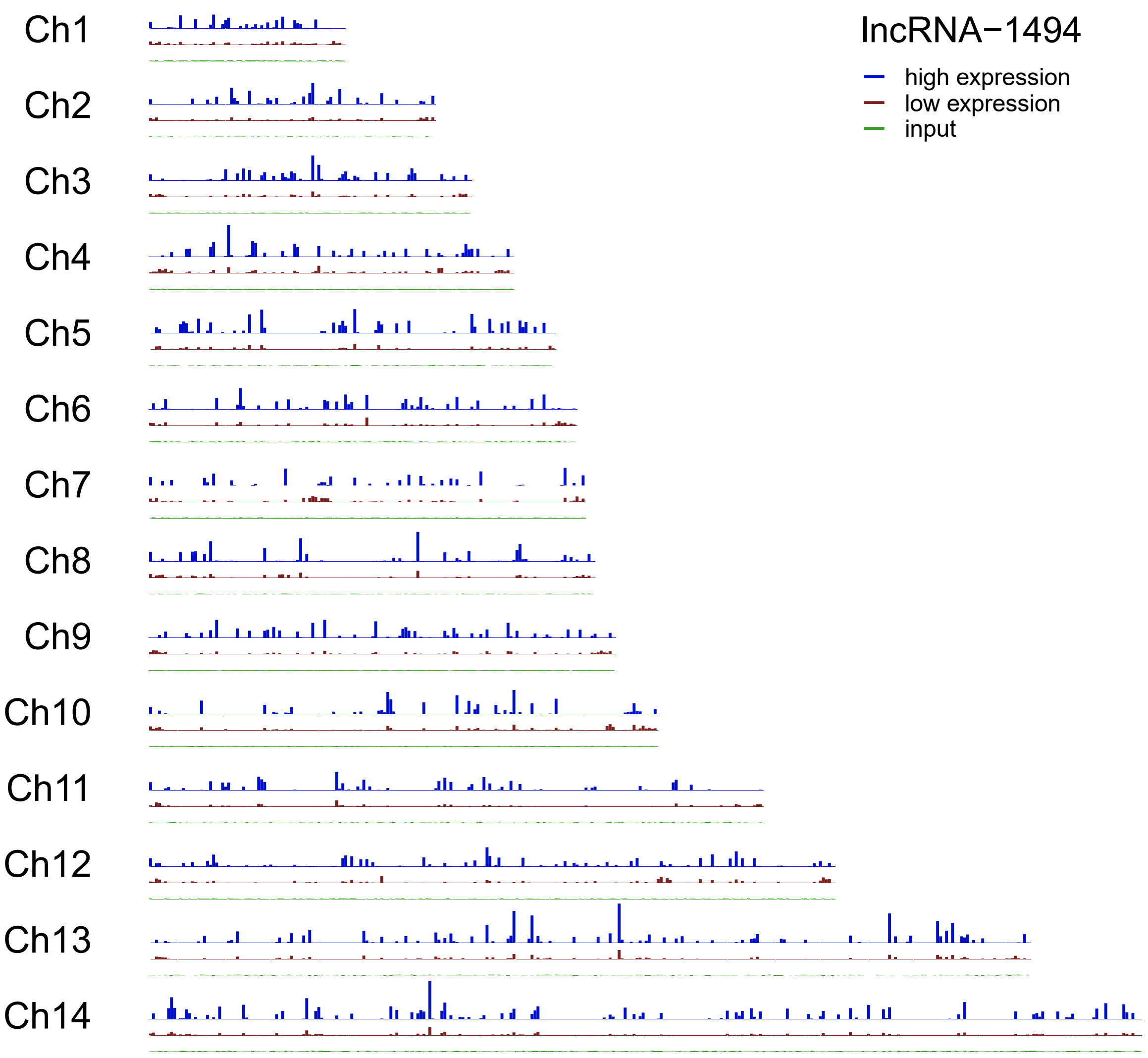

Fig. S4: (**a**) Genome wide ChIRP-seq signal for lncRNA-1494. All lncRNA libraries, including the input, were normalized by dividing by the numbers of mapped reads in each of them. For each nucleotide, the signal from the input library was subtracted from each of the ChIRP-seq libraries, and any negative value was replaced with a zero. Genome tracks are displayed for each chromosome (Ch).

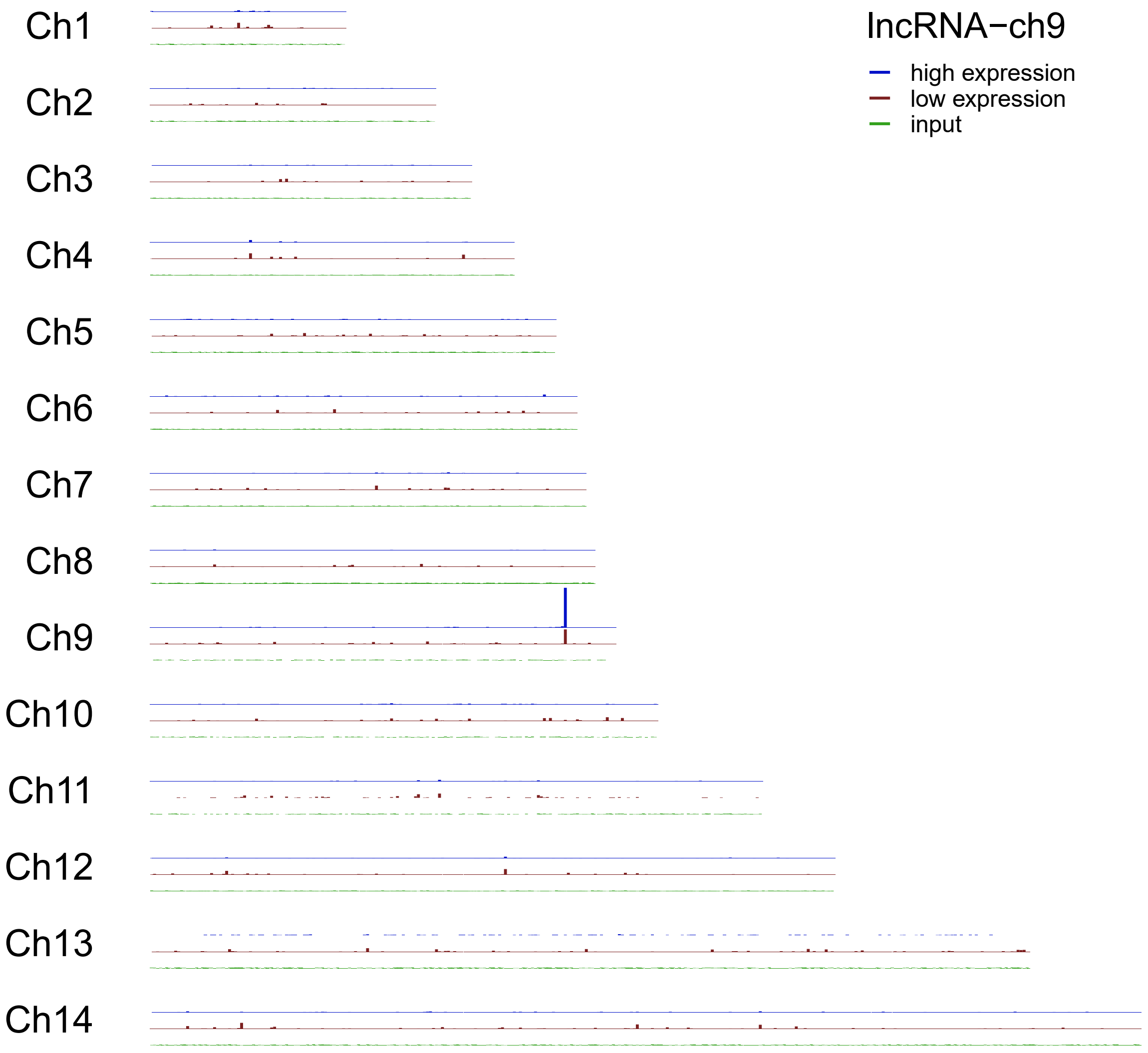

Fig. S4: (**a**) Genome wide ChIRP-seq signal for lncRNA-9. All lncRNA libraries, including the input, were normalized by dividing by the numbers of mapped reads in each of them. For each nucleotide, the signal from the input library was subtracted from each of the ChIRP-seq libraries, and any negative value was replaced with a zero. Genome tracks are displayed for each chromosome (Ch).

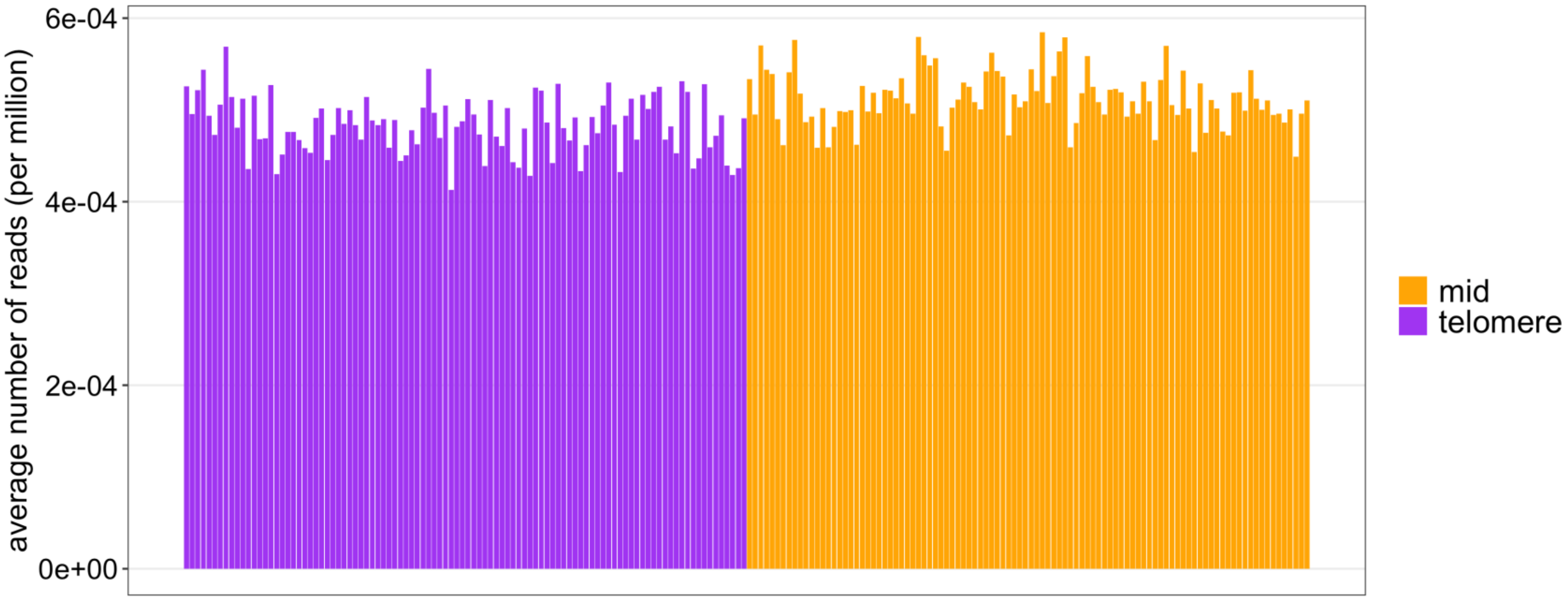

Fig. S4: (**b**) Coverage of various genomic regions detected in the input samples to demonstrate no potential artificial enrichment in the test samples. *Telomere* regions were defined as a 100,000 bp window at the end of each chromosome. *Middle* (mid) regions were defined as 100,000bp window centered at the locus half the distance from the centromere and the chromosome end. All Input signals were then aggregated over the 28 (2x14 chromosomes) *telomere* and *middle* windows and compared to the aggregated ChIRP-seq. Each sample was normalized by millions of reads. Overall, no depletion of the input signal was observed in the telomere regions.

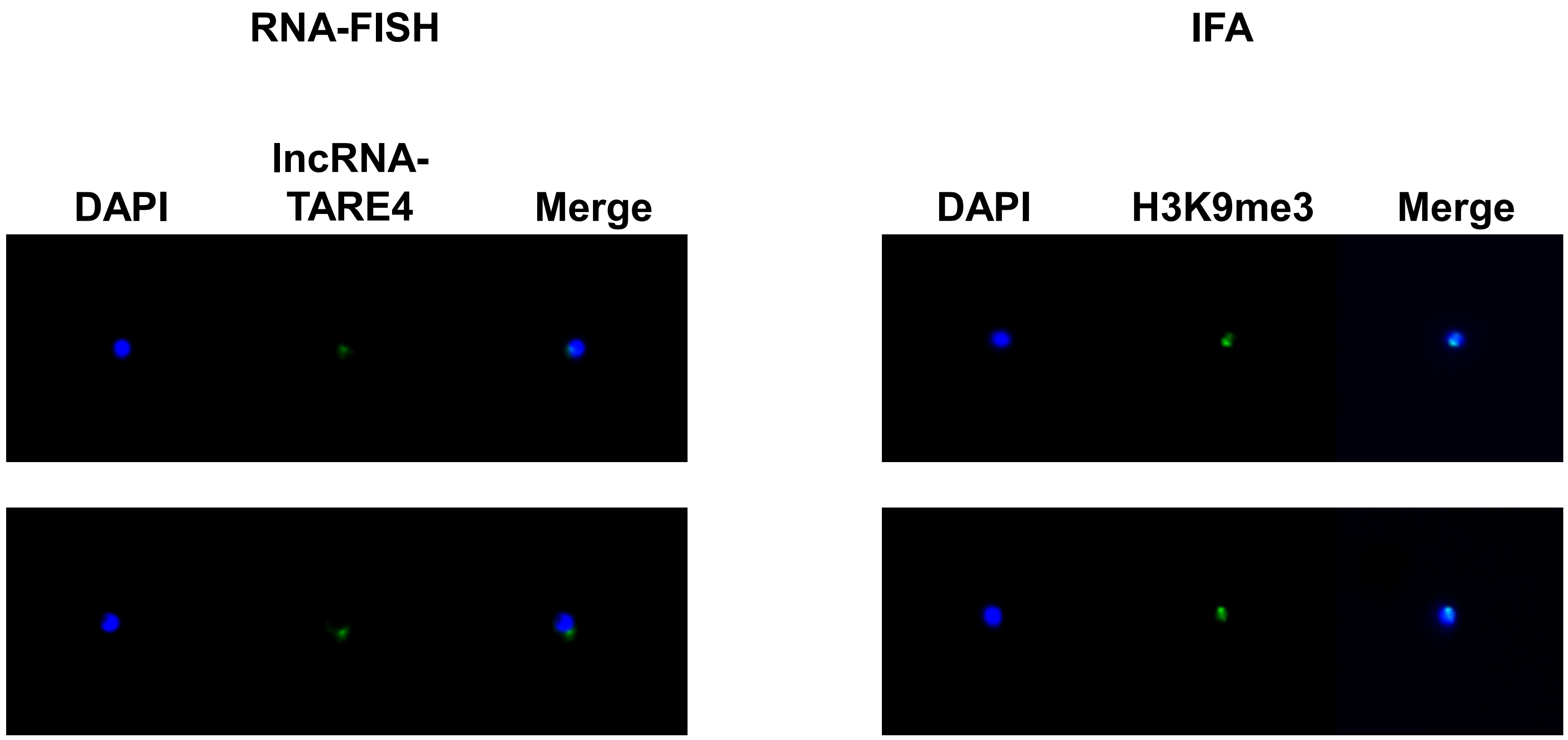

Fig. S5: Localization of lncRNA-TARE4 and H3K9me3. RNA-FISH immunofluorescence assays (IFA) experiments using lncRNA-TARE4 probes and anti-H3K9me3 antibody, respectively. The nuclear lncRNA-TARE4 and H3K9me3 mark colocalize with nuclei stained with DAPI in ring stage. Hybridization and IFA images are representative of approximately 15 stained parasites from two independent experiments.

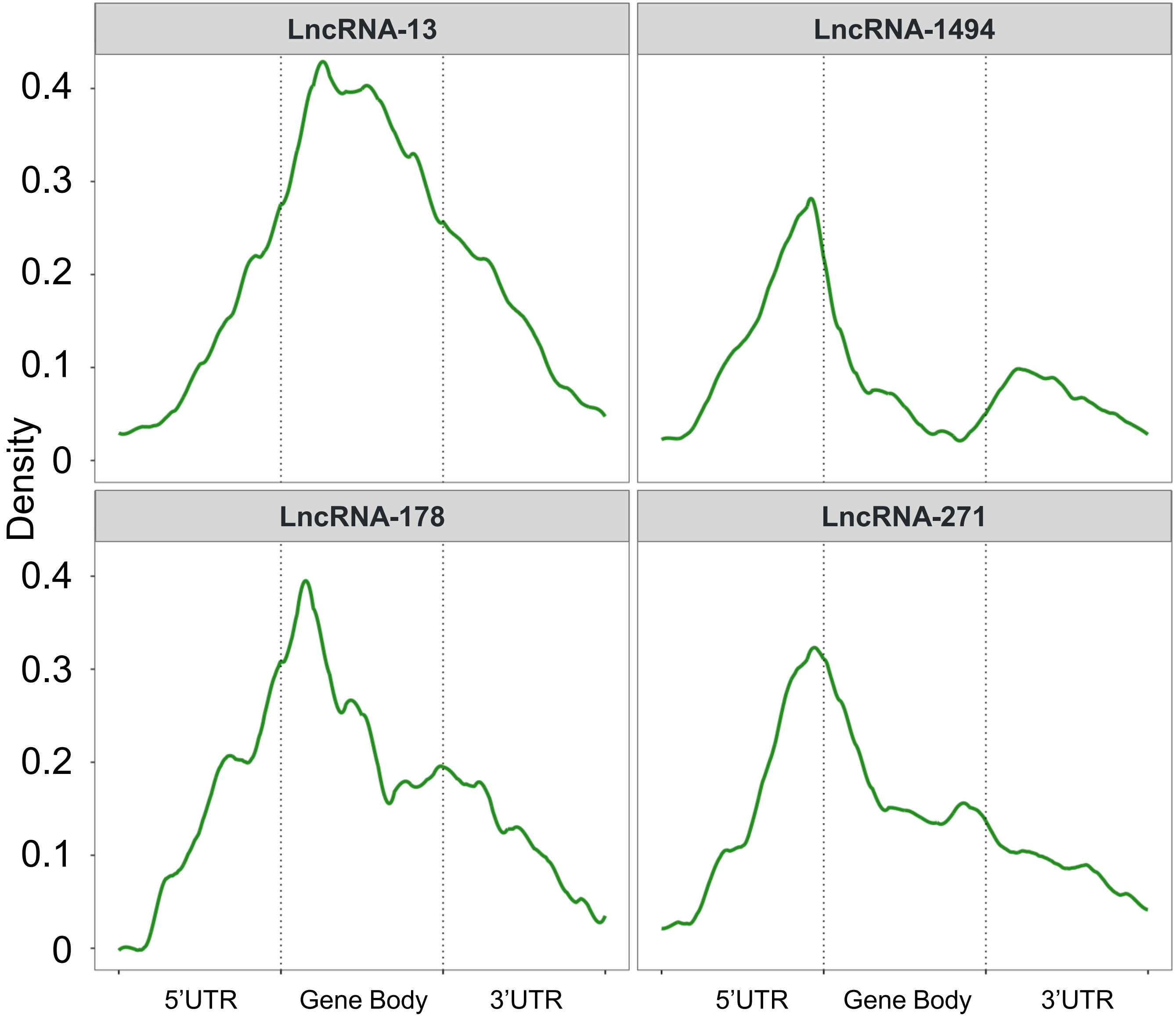

Fig. S6: Average of ChIRP-seq signals across all 5' and 3' UTRs and gene bodies. For a given lncRNA of interest we examined the regions of the genome that its corresponding peaks overlap. We extended a 1000bp window from the 5' and 3' ends of the nearest gene to each peak. We aggregated these genomic windows together with the gene body (normalized to 1000bp in order to account for different gene lengths) into a continuous 3000b window (x-axis). For each base position we plotted the density (the number of peaks overlapping the respective position divided by the total number of peaks) on the y-axis.

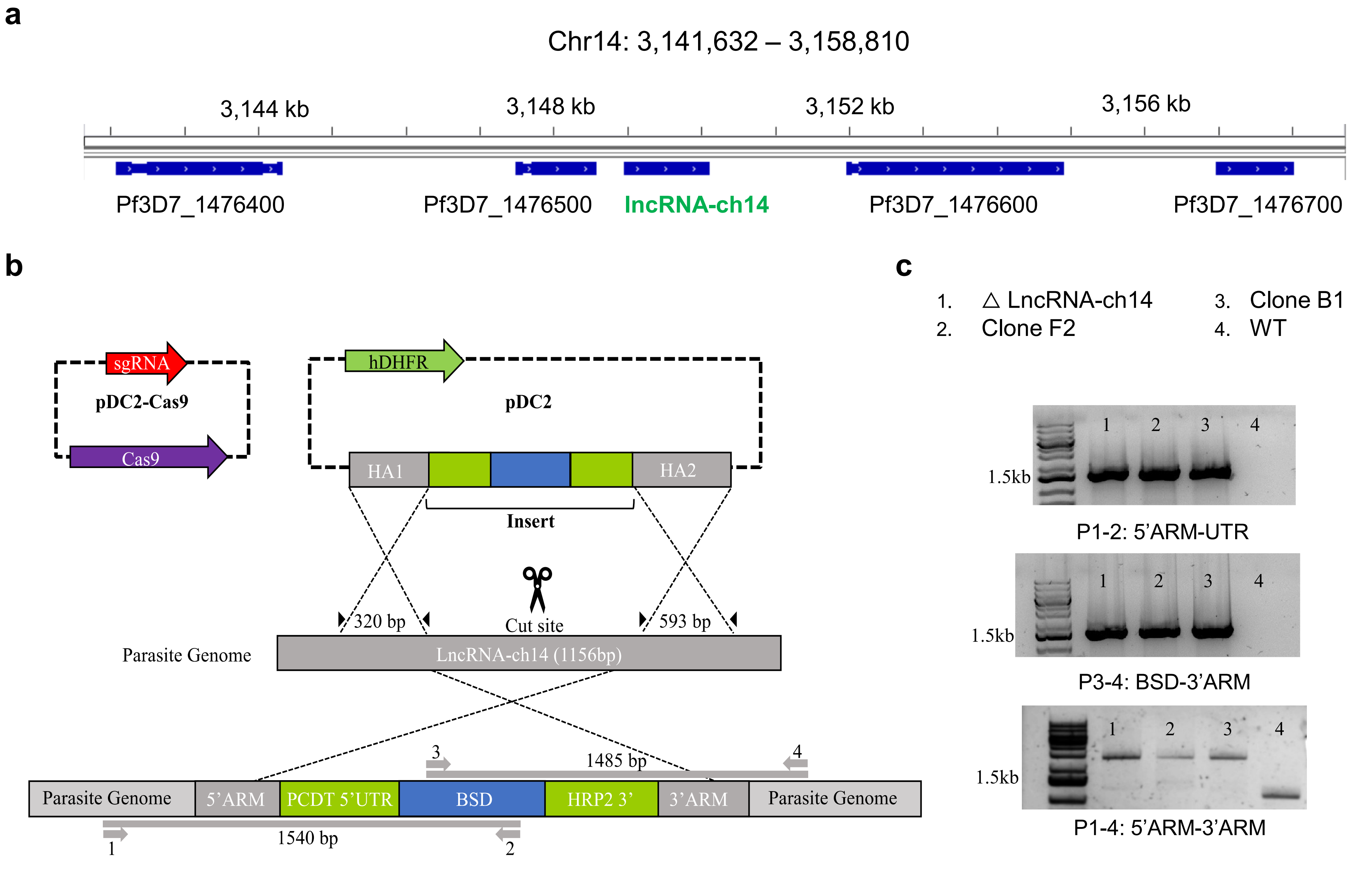

Fig. S7: LncRNA-ch14 disruption design. (**a**) Schematic representation of lncRNA-ch14 genomic region. **(b)** LncRNA-ch14 disruption strategy using the CRISPR- Cas9 tool through BSD^r insertion. Created with BioRender.com. (**c**) PCR Gel verification for lncRNA-ch14 disruption through PCR of 5'Arm insertion (Left), BSD^r and 3'Arm insertion (Middle) and entire lncRNA-ch14 segment (Right). The PCRs are representative of two independent experiments.

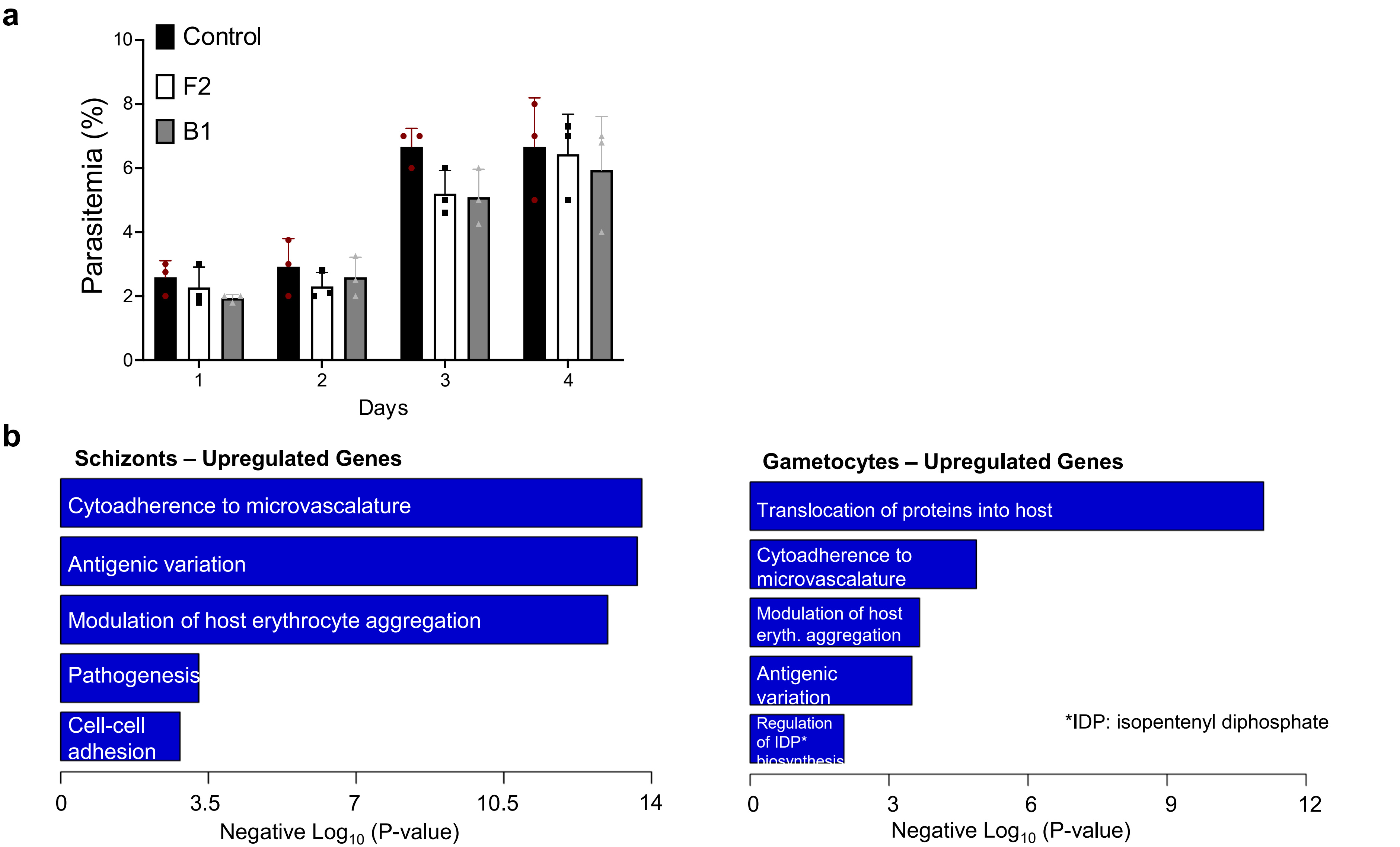

Fig. S8: Characterization of △lncRNA-ch14 line. (**a**) Asexual Stage Development. Parasitemia was assessed by Giemsa-stained blood smears. Three independent experiments were performed and data are presented as mean values +/- SD. Significance of the results was calculated using two-way RM ANOVA with multiple comparison Tukey's Test. (**b**) Gene Ontology enrichment analysis. Bar graph representations of selected Gene Ontology (GO) enrichment of upregulated genes between WT and △lncRNA-ch14 lines are presented by Log_{10} (P-value) (y-axis) in asexual mature (Left) and mature gametocyte (Right) stages.
