# Supplementary material for: Novel insights into the role of long non-coding RNA in the human malaria parasite, Plasmodium falciparum
Source: Nat Commun. 2023 Aug 22;14:5086. doi: 10.1038/s41467-023-40883-w (PMC10444892; doi:10.1038/s41467-023-40883-w)
Supplement: Supplementary file 2 — Description of additional supplementary files [file 41467_2023_40883_MOESM2_ESM.pdf]

Description of additional supplementary files

Supplementary data 1: Putative lncRNAs identified and investigated in this study. Chromosome, coordinates, and expression profile for all lncRNAs identified along with probe and primer sequences used to investigate several stage-specific lncRNAs.

1a: Mapping statistic for Nuclear and Cytoplasmic RNA-seq data.
1b: Putative lncRNAs identified in this study.
1c: Sequences of primers used for RT-PCR and the generation of RNA-FISH probes. The oligonucleotides were synthetized by IDT at 25 nmole scale.
1d: Number of foci detected by RNA-FISH.
1e: LncRNAs used for ChIRP-seq analysis.
1f: ChIRP-seq probes and sequences used for identifying lncRNA-chromatin interactions. The oligonucleotides were synthetized by Sigma at 100 nmole scale.
1g: lncRNA-TARE4 ChIRP-seq peak calling results. P-values (pval) were determined by negative binomial with local variance estimation [81.]
1h: lncRNA-13 ChIRP-seq peak calling results. P-values (pval) were determined by negative binomial with local variance estimation [81.]
1i: lncRNA-178 ChIRP-seq peak calling results. P-values (pval) were determined by negative binomial with local variance estimation [81.]
1j: lncRNA-271 ChIRP-seq peak calling results. P-values (pval) were determined by negative binomial with local variance estimation [81.]
1k: lncRNA-ch9 ChIRP-seq peak calling results. P-values (pval) were determined by negative binomial with local variance estimation [81.]
1l: lncRNA-ch14 ChIRP-seq peak calling results. P-values (pval) were determined by negative binomial with local variance estimation [81.]
1 : lncRNA-1494 ChIRP-seq peak calling results. P-values (pval) were determined by negative binomial with local variance estimation [81.]
1m: List of primers used for lncRNA-14 KO. The oligonucleotides were synthetized by IDT at 25 nmole scale.

Supplementary data 2: Whole genome sequencing results showing identified mutations in NF54 control and △lncRNA-ch14 transfected lines. The mutation in the *gdv1* gene essential for gametocyte formation exists only in the parental NF54 parasites highlighted in red.

Supplementary data 3: DEseq2 analysis of △lncRNA-ch14 clone compared to WT controls. Results for schizont and gametocyte stages are presented in two different sheets. The p-values were obtained by two-tailed Wald test and corrected for multiple testing using the Benjamini-Hochberg correction.
