# Supplementary material for: Novel insights into the role of long non-coding RNA in the human malaria parasite, Plasmodium falciparum
Source: Nat Commun. 2023 Aug 22;14:5086. doi: 10.1038/s41467-023-40883-w (PMC10444892; doi:10.1038/s41467-023-40883-w)
Supplement: Supplementary file 6 — Reporting Summary [file 41467_2023_40883_MOESM6_ESM.pdf]

nature portfolio

Corresponding author(s): Karine Le Roch

Last updated by author(s): Jul 19, 2023

Reporting Summary

Nature Portfolio wishes to improve the reproducibility of the work that we publish. This form provides structure for consistency and transparency in reporting. For further information on Nature Portfolio policies, see our Editorial Policies and the Editorial Policy Checklist.

Statistics

For all statistical analyses, confirm that the following items are present in the figure legend, table legend, main text, or Methods section.

n/a	Confirmed	
☐	☒	The exact sample size (*n*) for each experimental group/condition, given as a discrete number and unit of measurement
☐	☒	A statement on whether measurements were taken from distinct samples or whether the same sample was measured repeatedly
☐	☒	The statistical test(s) used AND whether they are one- or two-sided<br>*Only common tests should be described solely by name; describe more complex techniques in the Methods section.*
☒	☐	A description of all covariates tested
☐	☒	A description of any assumptions or corrections, such as tests of normality and adjustment for multiple comparisons
☐	☒	A full description of the statistical parameters including central tendency (e.g. means) or other basic estimates (e.g. regression coefficient) AND variation (e.g. standard deviation) or associated estimates of uncertainty (e.g. confidence intervals)
☐	☒	For null hypothesis testing, the test statistic (e.g. *F*, *t*, *r*) with confidence intervals, effect sizes, degrees of freedom and *P* value noted<br>*Give P values as exact values whenever suitable.*
☒	☐	For Bayesian analysis, information on the choice of priors and Markov chain Monte Carlo settings
☒	☐	For hierarchical and complex designs, identification of the appropriate level for tests and full reporting of outcomes
☐	☒	Estimates of effect sizes (e.g. Cohen's *d*, Pearson's *r*), indicating how they were calculated

Our web collection on statistics for biologists contains articles on many of the points above.

Software and code

Policy information about availability of computer code

Data collection	Image Lab software v5<br>Bio-Rad ChemiDoc MP Gel Imager<br>Chromium 10X v2 and v3 chemistry
Data analysis	ImageJ v1.53i<br>GraphPad Prism v9.1.2<br>GATK HaplotypeCaller v4.1.4.0<br>FastQC v0.11.8<br>CPAT, http://lilab.research.bcm.edu<br>R package Seurat v3.2.2<br>scmap-cell v1.8.0<br>liftOver Kent v427<br>CellRanger pipeline v3.0.0<br>R package EnhancedVolcano v1.18.0<br>R package ggplot2<br>DEseq2 v1.28.1<br>PicardTools v1.78<br>HISAT2 v2.2.1<br>BEDTools v2.25.0

PePr peak calling pipeline v1.1
Trimmomatic v0.39
Sickle v1.33
Bowtie2 v2.4.2 and v2.4.4
Samtools v 0.1.19-44428cd and v1.9
cufflinks v2.1.1
bwa v0.7.17
Python v3.73

For manuscripts utilizing custom algorithms or software that are central to the research but not yet described in published literature, software must be made available to editors and reviewers. We strongly encourage code deposition in a community repository (e.g. GitHub). See the Nature Portfolio guidelines for submitting code & software for further information.

Data

Policy information about availability of data

All manuscripts must include a data availability statement. This statement should provide the following information, where applicable:

- Accession codes, unique identifiers, or web links for publicly available datasets
- A description of any restrictions on data availability
- For clinical datasets or third party data, please ensure that the statement adheres to our policy

The GRO-seq data used in this study are available in the Gene Expression Omnibus database under accession code GSE85478 (https://www.ncbi.nlm.nih.gov/geo/query/acc.cgi?acc=GSE85478). The steady-state RNA-seq data used in this study are available the NCBI Sequence Read Archive under accession code SRP026367, SRS417027, SRS417268, and SRS417269 (https://www.ncbi.nlm.nih.gov/sra/?term=SRP026367, https://www.ncbi.nlm.nih.gov/sra/?term=SRS417027, https://www.ncbi.nlm.nih.gov/sra/?term=SRS417268, https://www.ncbi.nlm.nih.gov/sra/?term=SRS417269).
WGS, ChIRP-seq and RNA-seq data generated in this study (66 libraries) have been deposited in the NCBI Sequence Read Archive with accession PRJNA869073 and are available at https://www.ncbi.nlm.nih.gov/bioproject/PRJNA869073/. All other data generated in this study are provided in the Supplementary Information, Supplementary data and Source Data file.

Research involving human participants, their data, or biological material

Policy information about studies with human participants or human data. See also policy information about sex, gender (identity/presentation), and sexual orientation and race, ethnicity and racism.

Reporting on sex and gender	No human research participants were used in this study.
Reporting on race, ethnicity, or other socially relevant groupings	No human research participants were used in this study.
Population characteristics	No human research participants were used in this study.
Recruitment	No human research participants were used in this study.
Ethics oversight	No human research participants were used in this study.

Note that full information on the approval of the study protocol must also be provided in the manuscript.

Field-specific reporting

Please select the one below that is the best fit for your research. If you are not sure, read the appropriate sections before making your selection.

[x] Life sciences [ ] Behavioural & social sciences [ ] Ecological, evolutionary & environmental sciences

For a reference copy of the document with all sections, see nature.com/documents/nr-reporting-summary-flat.pdf

Life sciences study design

All studies must disclose on these points even when the disclosure is negative.

Sample size	Sample sizes were chosen according to standards in the field and based on our previous experience with similar experiments. For aseuxal and gametocyte parasitemia, at least 10 fields were counted (300-500 red blood cells) (PMID: 35277503 and 36241929). For male:female ratio quantification, at least 500 mature gametocytes were counted. For exflagellation assays at least 10 fields per condition were evaluated (PMID: 36798298 and 36241929). For oocyst and sporozoite experimentations, roughly 15-20 Anopheles stephensi mosquitoes were used for each assay conducted (PMID: 32716382 and 36798298). Sample size and statistical analysis are indicated in the Methods section.
Data exclusions	No data was excluded.
Replication	All attempts at replication were successful. Spearman correlation coefficient was performed to validate the reproducibility of RNA-seq and all mosquito stage quantification assays were performed in duplicates. Mature gametocyte quantification assays were performed at least in duplicate, ChIRP-seq assays were performed in duplicate. Parasitemia, phenotypic analysis, immunoblots, were done in replicate in at least

	two independent experiments.
Randomization	Assignment of parasites for cellular fractionation, ChIRP-seq, RNA-FISH were randomized in the study.
Blinding	The parasitemia of asexual and mature gametocyte stages and phenotypic analyzes were calculated blind and validated by a third party. Male:female ratio, exflagellation, ocysts and sporozoites counts were not blinded for our collaborators.

Reporting for specific materials, systems and methods

We require information from authors about some types of materials, experimental systems and methods used in many studies. Here, indicate whether each material, system or method listed is relevant to your study. If you are not sure if a list item applies to your research, read the appropriate section before selecting a response.

Materials & experimental systems

n/a	Involved in the study
☐	☒ Antibodies
☐	☒ Eukaryotic cell lines
☒	☐ Palaeontology and archaeology
☐	☒ Animals and other organisms
☒	☐ Clinical data
☒	☐ Dual use research of concern
☒	☐ Plants

Methods

n/a	Involved in the study
☒	☐ ChIP-seq
☒	☐ Flow cytometry
☒	☐ MRI-based neuroimaging

Antibodies

Antibodies used	Primary antibody for Immunoblot:<br>Anti-H3 antibody (Abcam; ab1791 lot GR158095-1) at 1:3000 dilution.<br>Anti-Plasmodium Aldolase (Abcam, ab207494) at 1:1000 dilution.<br><br>Secondary antibodies for immunoblot: Goat Anti-Rabbit IgG HRP Conjugate (Bio-Rad; 1706515) at 1:25000 dilution.<br><br>Primary antibody for IFA:<br>Anti-Histone H3 (tri methyl K9) antibody [6F12-H4] (Abcam, ab184677) at 1:500 dilution.<br><br>Secondary antibody for IFA:<br>Goat anti-Mouse Alexa Fluor 488 (Invitrogen, A11001, lot 2051236) at 1:2000 dilution.
Validation	Anti-H3 antibody (Abcam; ab1791) was validated for immunoblot by Abcam and previously validated in ChIP-seq in P. falciparum based on this publication (https://www.nature.com/articles/srep31965).<br><br>Anti-Plasmodium Aldolase (Abcam, ab207494) is described by Abcam as: Suitable for: WB, ELISA and Reacts with: Plasmodium falciparum aldolase. It was previously validated by WB in P. falciparum (https://www.nature.com/articles/s41467-022-28981-7)<br><br>Anti-H3K9me3 is described by Abcam as suitable for immunohistochemistry and was previously validated by IFA (https://link.springer.com/protocol/10.1007/978-1-0716-0763-3_3).

Eukaryotic cell lines

Policy information about cell lines and Sex and Gender in Research

Cell line source(s)	Plasmodium falciparum NF54 strain was provided by the Malaria Research and Reference Reagent Resource Center (MR4)
Authentication	The lines were analyzed and validated by whole-genome sequencing
Mycoplasma contamination	Not tested
Commonly misidentified lines (See ICLAC register)	No commonly misidentified lines were used in this study

Animals and other research organisms

Policy information about studies involving animals; ARRIVE guidelines recommended for reporting animal research, and Sex and Gender in Research

Laboratory animals	No laboratory animals were used.
Wild animals	No wild animals were used in this study.
Reporting on sex	No animals were used in this study.
Field-collected samples	No field collected samples were used in this study.
Ethics oversight	No ethics oversight in this study.

Note that full information on the approval of the study protocol must also be provided in the manuscript.
